# *‘A Dead Person Cannot Carry a Dead Person’*: Health, Social Support and Language Learning Among Syrian Refugees in Norway

**DOI:** 10.3390/ijerph23010047

**Published:** 2025-12-29

**Authors:** Ayan B. Sheikh-Mohamed, Esperanza Diaz, Melanie Straiton, Arnfinn Jomar Andersen

**Affiliations:** 1 Department of Global Public Health and Primary Care, Faculty of Medicine, University of Bergen, 5020 Bergen, Norway; esperanza.diaz@uib.no; 2 Norwegian Centre for Violence and Traumatic Stress Studies, 0484 Oslo, Norway; arnfinn.andersen@nkvts.no; 3 Norwegian Institute of Public Health, 0456 Oslo, Norway; melanielindsay.straiton@fhi.no

**Keywords:** refugees, emigration and immigration, social determinants of health, language development, social participation

## Abstract

**Highlights:**

**Public health relevance—How does this work relate to a public health issue?**

- Examines how language learning and health are interconnected in the daily lives of Syrian refugees.
- Highlights social participation as an accessible factor that supports both language development and health.

**Public health significance—Why is this work of significance to public health?**

- Centers refugees’ lived experiences to better understand the links between health, everyday support, and language learning.
- Shows that language learning is shaped by social determinants of health, including power asymmetry and social relations.

**Public health implications—What are the key implications or messages for practitioners, policy makers and/or researchers in public health?**

- Suggests that integration and health policy should support community-based arenas beyond classroom learning.
- Emphasizes that inclusive, relational settings promote both participation and health equity among refugees.

**Abstract:**

Second language acquisition (SLA) is critical for refugee integration and a determinant of health and health care access. Although numerous studies have examined language barriers and health communication, the reciprocal relationship between health and second language acquisition remains underexplored in public health research. This qualitative study draws on interviews with twenty Syrian refugees (nine men and eleven women, aged 22–65) resettled in Norway. Data were collected through semi-structured interviews and analysed using reflexive thematic analysis. Two overarching themes were identified: (1) Learning under strain: health problems and post-migratory stressors constrained SLA; and (2) Relational support: reciprocal interactions with neighbours, colleagues, and volunteers enabled both language learning and functional health. These social arenas acted as low-threshold, health-promoting settings that mitigated isolation and strengthened belonging. The study highlights that language operates as a social determinant of health: inclusive, relational spaces facilitate both SLA and health by enhancing communicative participation and access to care. Refugee integration policy should therefore support accessible community spaces outside formal education to strengthen social inclusion, health literacy and refugees’ ability to navigate health and welfare services.

## 1. Introduction

Newly arrived refugees in high-income countries navigate a demanding transition from forced migration to settlement in a new and unfamiliar context. This period is often marked by migratory stressors, which are known to affect mental health [1,2,3]. Learning the language of the receiving country is one of the many post-migratory challenges refugees face [4]. At the same time, language proficiency is a prerequisite for economic, political, social, and cultural integration [5,6]. Research shows that mental health improved among Syrian refugees from transit to early resettlement [7]. However, this initial improvement in the first few months after arrival, the so-called “honeymoon period”, appears to be short-lived, and resettlement generally involves substantial stressors including acculturative stress, discrimination and challenges in accessing services [8,9,10]. Such strain may affect both health and opportunities for second language acquisition (SLA).

Beyond stress being a common underlying factor, a number of studies indicate a relationship between SLA and health in migrants. Although there is a large body of literature on the association between language acquisition and mental health in refugees [11,12,13,14,15,16], there are fewer studies considering the relationship between somatic health and SLA. Here we use the term “somatic health” to refer to bodily symptoms and physical complaints, including pain and other common manifestations of stress. Although rarely investigated, some studies have explored this connection. For example, a retrospective study of electronic health records of almost 80 thousand health care visits in the US found that poor SLA was associated with an increased risk of reporting pain in migrants [17]. A cross-sectional, register-based study involving 116 Syrian migrants in Germany found an inverse relationship between German language proficiency and somatic complaints such as chest pain, nausea, and shortness of breath, measured using the PHQ-15 scale [18]. A longitudinal study of humanitarian migrants in Australia found a positive and significant relationship between language proficiency and self-reported physical health [19]. Taken together, these studies indicate that SLA and health are intertwined. At the same time refugees’ health challenges may themselves limit participation in language learning. However, the underlying mechanisms remain unclear, particularly how health challenges may constrain or be mitigated in learning contexts.

In that regard, the Norwegian context can be particularly relevant, given its highly regulated language training system. Language training of refugees is not just encouraged; it is legally mandated, with non-compliance potentially leading to financial penalties [20]. Adult refugees have to participate in a mandatory two-year Introduction Program [21]. Participants in the program are entitled to free language training and social studies along with introductory financial assistance to provide support during the training period [21]. Valid absence from the Introduction Program includes illness that must be confirmed by health personnel [22]. Citizenship, often regarded as the final marker of belonging and recognition, hinges on passing both language and civics tests [23].

In this study, we focus on Syrian refugees residing in Norway, as they constitute one of the largest refugee groups in the country [24]. Following the onset of the Civil War, nearly 26,000 individuals were resettled in Norway between 2015 and 2017 [25]. Syrian refugees have typically endured violence, displacement and trauma before fleeing [26]. Upon arriving in Europe, welcome was framed by conditions that implied dependence and normative expectations of gratitude and assimilation [27,28]. Drawing on Derrida’s conceptualisation of hospitality [29], these conditions can be understood as reflecting the conditional nature of hospitality and how it is embedded in power relations. Consequently, integration policies can operate as a regime of obligations that require refugees to prove their worth through, for example, language acquisition [30]. In the Norwegian context, caseworkers in welfare services and other public bodies exercise this gatekeeping role, shaping refugees’ access to resources within an integration framework that demands conformity to culturally specific values of self-reliance and egalitarianism [31]. This can lead to a sense of powerlessness, which, according to the Commission on Social Determinants of Health (SDH), is a root cause of health inequity [32]. Furthermore, as Sir Michael Marmot notes, lack of control and limited social engagement can trigger chronic stress, activating pathways that link social inequity and biological health outcomes [33]. To understand the implications of lacking control, the work of German-American historian and philosopher Hannah Arendt offers a useful framework. She emphasizes that power is generated through “acting in concert” [34]. Drawing on Arendt’s concept of power, Habermas interprets it as communicatively constituted, where individuals show their subjectivity while establishing others as responsible equals able to find common ground, creating symmetry and power [35]. Conversely, feelings of powerlessness can potentially result in adverse health outcomes [36].

These insights illustrate how power dynamics can manifest in health, framing the challenges faced by Syrian refugees. Research in Nordic countries indicates that refugees experience higher rates of mental health problems than other migrant groups and the general population [37,38,39]. Studies on Syrian refugees in Norway highlighted a high prevalence of chronic pain [7,40,41] and pointed to a connection between mental health disorders and chronic pain [40,41]. One of the studies also suggests that many Syrian refugees with non-communicable diseases in Norway do not receive appropriate medication, with headache and musculoskeletal problems being the most frequently reported conditions [7]. Against this backdrop, Syrian refugees constitute an important lens for examining how social support, SLA and health intersect.

In line with Huber et al., we understand health as “the ability to adapt and self-manage in the face of social, physical, and emotional challenges” [42]. This shifts the focus from the absence of disease to the dynamic interplay of opportunities and limitations. Furthermore, consistent with the World Health Organization’s framework for SDH [43], we view language as a determinant that shapes access to education, communication, and social inclusion, thereby influencing health trajectories. In this framing, language is not only a communicative tool but a social determinant of health: it shapes access to health care systems and opens pathways to social networks that provide support. Vygotsky’s Sociocultural Learning Theory [44] highlights how interaction and social support can mediate the effects of SDH. From this perspective, learning is a socially mediated process in which guidance within the Zone of Proximal Development enables learners to actively co-construct knowledge [44]. Taken together, these perspectives suggest that SLA can be understood not simply as a cognitive task, but as a socially and politically situated process that is deeply entangled with health.

### Aim of the Study

This paper explores how health and social conditions intersect with SLA among Syrian refugees in Norway and asks:

1. How do health challenges interact with refugees’ opportunities for language learning?
2. How can social relations and everyday interactions support SLA in the context of health-related vulnerability?

## 2. Methodology

This qualitative study is part of the Integration for Health study and a continuation of two previous studies: 1) CHART study (Changing Health and health care needs Along the Syrian Refugees’ Trajectories to Norway) and 2) REFUGE study (Resettlement in Uprooted Groups Explored).

CHART followed Syrian quota refugees from Lebanon to Norway, documenting changes in quality of life, healthcare utilization patterns, and the burden of chronic pain and mental health problems [7,41,45,46]. REFUGE examined mental health outcomes and associated risk factors among Syrian refugees resettled in Norway, revealing substantial psychological distress and demonstrating a dose–response relationship between trauma exposure and mental health symptoms [38]

### 2.1. Participants and Recruitment

The sample consists of 20 adult Syrian refugees (11 women, 9 men), aged between 22 and 65 years, who had arrived in Norway between 2015 and 2017. Participant demographics are presented in Table 1. Recruitment combined snowball sampling via professional and community networks in Oslo and Bergen followed by purposeful sampling to ensure diversity in gender, age, education, and occupational background [47]. At the time of first interviews (March–May 2023), all had lived in Norway for 6–8 years, providing them with experience of navigating the recipient society and finding their place. Inclusion criteria were Syrian origin, adult age, resettlement in Norway between 2015 and 2017, and willingness to participate in in-depth interviews; language proficiency in Norwegian was not an inclusion criterion.

### 2.2. Data Collection

We conducted two rounds of semi-structured, individual interviews. The first round (n = 20) was conducted between March and May 2023 and lasted 50–150 min, with an average of one hour. Interviews followed a guide covering broad themes such as Daily Life in Norway, Social Networks, Health and Integration, and Future Outlook. This allowed participants to reflect on their everyday lives while leaving room for developing insights.

A second round of follow-up interviews were conducted with 10 participants between December 2023 and February 2025. These interviews were typically shorter, an average of 20 min, and more targeted. They focused on developments since the first interview and on themes that had developed as salient in the initial analysis, including health challenges and SLA. The time between first and second interviews ranged from nine to 20 months. Follow-ups thus added a longitudinal dimension and enabled participants to elaborate on earlier narratives or discuss new developments, such as employment, social participation, and health status.

Most interviews were conducted face-to-face. Three follow-ups were conducted by encrypted videoconference at participants’ request. In two of these cases, a participant who had previously taken part in the study facilitated contact with peers who wished to contribute and acted as an interpreter during the video calls.

Twelve participants required interpreter assistance. All participants were offered the option of interpreter assistance. Some chose to use an interpreter despite having conversational Norwegian skills, to express complex experiences related to health, emotions and social relations in their first language. The continued use of interpreters reflects variation in language acquisition trajectories and aligns with the study’s focus on how health and social conditions shape opportunities for SLA. In the first round, nine of these were supported by a professional interpreter, two by another interpreter, and one by an Arabic-speaking colleague in the research group. In the second round, five participants again required interpretation: three were assisted by the main interpreter, and in two cases a fellow participant acted as interpreter. Both professional interpreters introduced themselves at the onset, explained their neutral role, and assured participants of confidentiality. The use of a participant as interpreter in the second round was not planned but was accepted to respect participants’ interest in sharing their experiences, and their relevance for the study.

All interviews were audio-recorded using the GDPR-compliant [48] app called Diktafon. Data were securely transferred and transcribed verbatim in Norwegian.

### 2.3. Data Analysis

In the analysis, we were guided by reflexive thematic analysis following Braun and Clarke’s six-phase model [49]. Transcripts from both rounds of interviews were manually coded using Word. Initial open coding was inductive and captured a wide range of experiences around being new in a different society. As the follow-up interviews progressed, coding became more focused, engaging explicitly with the intersection of health status and SLA. Because follow-up interviews were informed by participants’ earlier narratives, not all individuals were asked identical questions. This adaptive approach was consistent with reflexive thematic analysis, where developing insights can guide subsequent data collection.

The analytic process was iterative and theory-informed. After initial inductive coding, Krashen’s Affective Filter Hypothesis [50] appeared relevant for understanding how distress might hinder learning. However, this framework conceptualizes barriers primarily on the individual level and did not capture the contextual factors enough. Therefore, it was not taken forward. Vygotsky’s sociocultural learning theory and the concept of the Zone of Proximal Development [44] provided a more suitable lens for interpreting how trust-based interaction enabled learning despite health-related challenges. Arendt’s notion of communicative power [35] further illuminated how reciprocity and recognition countered experiences of powerlessness, while SDH perspectives could explain how inequality could become embodied, with somatic health challenges intersecting with language learning. These frameworks guided a dialogue between data and theory, situating the analysis within participants’ lived health and integration trajectories.

### 2.4. Ethical Considerations

The study was waived by the Regional Committee for Medical and Health Research Ethics (REK nr 564018). Participants received oral and written information, and consent was recorded before interviews started. They were reminded of their right to withdraw at any time without consequences. Names have been altered, and some demographic details where necessary, to protect anonymity, while avoiding unnecessary erasure.

All interviews were conducted by the lead researcher, who has professional experience in clinical healthcare and a refugee background, though not from Syria. This positioning offered both proximity and distance: an understanding of structural conditions relevant for refugees in Norway, while not sharing participants’ specific cultural and linguistic background.

Practical support was offered when appropriate, including information about psychosocial services. While some interviews were emotionally charged, participants did not report re-traumatisation; instead, several described the conversations as relieving.

## 3. Results

Through an iterative analytical process, two main themes were generated. While the analysis draws on accounts from all 20 participants, we present extended extracts from a subset. These were selected because they capture recurring patterns across the dataset, while also representing variation in age, gender, and health status. In this way, the voices highlighted here are not presented as exceptional, but as illustrative of broader shared meanings within the two themes.

The themes are as follows:

1. Learning under strain: Health challenges and SLA;
2. Relational Support to Language Learning.

### 3.1. Learning Under Strain: Health Challenges and SLA

The experiences of refugees learning a new language are potentially shaped by contexts of powerlessness, and by various psychological, social, structural and somatic health challenges. Participants in this study carry with them a profound sense of loss, having been deprived of family members, homes and parts of their identity. Many crossed country borders in the hands of smugglers, their lives dependent on others’ control; some arrived in overcrowded rubber boats, while others endured discrimination and deprivation in transit countries. These experiences accompanied them into a receiving society that was multifaceted, including both hostility and welcomes. A central theme, with different facets, was that distress negatively influenced participants’ ability to engage in language learning.

#### 3.1.1. Institutional Barriers

Interactions with institutions intended to provide care, welfare or education were at times marked by powerlessness or mistrust. These dynamics did not only arise from the interactions themselves, but also from participants’ interpretations and earlier experiences, shaping how institutions were perceived and navigated.

Zara, a married mother in her late twenties, developed symptoms of depression following her arrival, but avoided healthcare out of fear that disclosure might result in her son being taken away:

*… But not tell anyone, especially to the doctor and such, so I couldn’t… I didn’t have… We didn’t know much information about the country and such. Now we know that they don’t take children if you get depressed or have some problems… They try to help. But at that time, it wasn’t easy to understand that.*

Her silence illustrates how mistrust, rooted in uncertainty and limited knowledge, turned depression into an unspoken barrier to learning.

Lina, in her early twenties, felt repeatedly overlooked by teachers in class, perceived by her as racism:

*I experience it [racism] almost every day. My teachers, they’re like… you know in class… and the teacher asks a question, and I raise my hand, want to answer what she asked. She looks at the other Norwegians, she does not look at me. She says I will take you afterwards and she always forgets me…*

Lina’s experience of exclusion and invisibility undermined her trust and confidence in the classroom. Feeling excluded not only restricted her opportunities to practice Norwegian language, but also reflected power imbalances that, over time, could become embodied stress with consequences for learning.

Ibrahim, a married father of five, caring for his chronically ill wife, could not fulfil attendance requirements and ultimately failed the language and civics test required for citizenship. His case illustrates how structural expectations collided with caregiving responsibilities, with consequences for rights and belonging.

These examples show how institutional barriers were not fixed properties of systems alone, but emerged in the interplay between participants’ own fears, histories and interpretations. From Arendt’s perspective [35], such dynamics could constrain the possibility of communicative actions between equals, narrowing the spaces where power, dignity and language learning might otherwise have been cultivated.

#### 3.1.2. Psychosocial Strains

Traumatic experiences before and during migration shaped how several participants engaged with language learning. Painful memories of armed conflict, loss and displacement often resurfaced after arrival. Ahmed, 27, said: *‘Reflections of the war come later,’* describing how trauma caught up with him once in safety. Habiba, a widow in her sixties who had lost her children during migration, reported, *‘Before, in my home country, I went to school, I worked, I had good mental health. But after my children went missing, and I became older, it is difficult for me to learn the language.’* Her persistent grief shows how loss can become embodied; consuming resources needed for SLA. Another participant, Hawa, a married mother in her fifties, described how loud noises, such as slamming of doors, triggered fear reactions. These examples illustrate how trauma can resurface in new contexts, diverting attention from learning.

For many participants, early resettlement period was marked by social isolation, amplifying distress. Ibrahim shared a well-known Arabic proverb—*‘Paradise without people means nothing’*—conveying how even a safe environment can feel empty without meaningful social bonds. This proverb illustrates more than emotional loneliness: It captures the absence of guided support in everyday life. Mawra, a divorcee in her forties, recalled: *‘… the loneliness that I felt here in Norway the first period. The first year I thought a lot about going back. That year I was alone most of the time, I was often depressed, I was inside a lot…*’. Such accounts illustrate how the absence of familiar networks could deepen loneliness and depression.

Psychosocial strain such as grief, trauma and loss was not only emotionally heavy but could undermined the interactive process needed for learning. In Vygotsky’s term, isolation cuts off access to learning with support within the Zone of Proximal Development, which is the zone between what a learner can independently achieve and what can be achieved with guidance [44]. Adding Arendt’s view, these burdens were not only internal, reflecting how spaces for mutuality were limited [35].

#### 3.1.3. Embodied Health Challenges

Participants described physical conditions that could impair their ability to engage in language learning. Ahmed linked constant worry for his family to overeating, obesity, and sleep apnoea, showing how emotional distress manifested physically. Others reported pain and sensory difficulties that disrupted their participation in classroom activities. Hawa explained:

*Yes, I had so much pain. In my back. And I couldn’t hear… And it affected me when I sat in school and could not hear what the teacher was saying… I felt so bad. Why can’t I do this? I was angry with myself*

Her hearing problems reduced learning ability, and her self-blame exacerbated feelings of inadequacy.

Mawra, in her forties, described constant fatigue, headaches, sleep apnoea, thyroid disease and weight gain: ‘*I was constantly tired, exhausted… I had headaches, I also woke up with headache also because I had sleep apnoea, without knowing what it was…And then the thyroid disease, the overweight’*. Her undiagnosed and untreated conditions prolonged and delayed her participation in language learning, illustrating how limited access to timely healthcare could add to learning barriers.

Layal, in her thirties, reported nerve damage from earlier medical treatments, stating, *‘Yes, and because of the treatment I have had some damage to my nerves. So, I have this ringing in my ears. All the time. Some letters I cannot hear’*. Her sensory impairment, she explained, reduced her ability to follow teaching and fully participate in the classroom.

These depictions demonstrate that somatic health challenges are not necessarily only isolated medical complaints but were intertwined with social conditions. This is in line with Sir Marmot’s perspective that lack of control and chronic stress can become biologically embedded [33], potentially manifesting as pain, fatigue or sensory impairment. The persistence of untreated conditions can further reflect structural determinants of health [43], including barriers in healthcare access and delayed diagnoses.

#### 3.1.4. Summary of Constraints

Structural constraints, such as rigid program demands and mistrust of institutions, could operate as negative social determinants of health and deepen feelings of powerlessness. Psychosocial strains, including trauma, loss and isolation, could weaken the reciprocity and recognition Vygotsky and Arendt view as prerequisites for learning from each other [35,44]. Embodied health challenges, in turn, demonstrated how stress and inequality could manifest somatically [33]. These interlinked processes reveal health and power as core to understanding participants’ language learning trajectories. Some participants, however, resisted this downward spiral. Their experiences highlight the facilitating role of social interaction and support, which we turn to in the next theme.

### 3.2. Relational Support to Language Learning

Several participants were able to acquire Norwegian language despite chronic pain, depression or sleep disturbances. Important for this learning was the presence of supportive relationships with members of the majority population. Such interactions, whether in workplaces, neighbourhoods or volunteer groups, created opportunities to practice language. Reciprocity and trust turned everyday encounters into learning moments, in line with Vygotsky’s account of guided support in learning [44]. Even with ongoing health difficulties, belonging in meaningful activities and relationships facilitated continued SLA. Importantly, a minimum of proficiency was often required to enter such exchanges, but once begun, interaction itself generated further learning and strengthened belonging. This theme of relational support as a pathway to language learning had different facets, to be presented next.

#### 3.2.1. Support Through Practical Activities

Participants often practiced Norwegian through everyday activities with trusted others. These shared practices became zones of learning, combining meaningful tasks with opportunities to rehearse the language in safe environments. Marwan, a father in his forties, was going through a difficult divorce and a depression that later required hospitalization. Despite this, his Norwegian skills quickly reached near-fluency. He credited an older colleague who supported him outside work through hiking and gym visits:

*And he was very kind and very helpful. He helped me with many important things, not only in my work. He helped me open a restaurant. And create a proprietorship… And what are the rules… Yeah, so he supported me in so many ways, not only in school*

These activity-based conversations provided linguistic input tied to concrete tasks and future goals, shifting identity from patient in crisis to competent colleague and aspiring business owner.

Lina described how neighbours provided her with both practical and emotional support:

*‘I am lucky because I live with good neighbours, they’re Norwegian neighbours. Very social… And they always greet me. They help me when I have difficulties.’* The father of the family regularly assisted with schoolwork: *‘He helps me when I have a presentation, or a difficult task that I cannot solve’.* Through these guided steps, challenging assignments became manageable, illustrating Vygotsky’s point that learning advances through supported interaction. These interactions built her confidence, creating a trustful environment that her teacher at school did not provide.

Other examples included playing football and learning to drive, activities that provided natural opportunities for language learning, aligning with Vygotsky’s theory, which emphasizes how learning develops through interactions with more knowledgeable others [44]. Practical activities thus fostered a sense of belonging, provided recognition, and facilitated learning, even in the presence of health-related struggles.

#### 3.2.2. Establishing Social Networks: Reciprocity

Reciprocal interactions fostered greater equality and created conditions for learning. Habiba, who otherwise mostly lived in grief and isolation, attended a women’s group twice a week. Despite limited language skills, she could practice through shared cooking: *‘We cook together, we eat together and also learn a little Norwegian. There are both Arabs and Norwegians there’*. Her expertise in cooking allowed her to give something back, balancing power relations and creating reciprocity.

Ahmed also stressed the importance of language in building networks, explaining, ‘*It [language] helps to gain networks, if you don’t have others to talk to with the same mother tongue. It [language] helps to have contacts…’* Through volunteer activities he formed numerous relationships with Norwegians, gaining practical, emotional and linguistic support. Despite symptoms of PTSD, obesity, and sleep disorders, he contributed actively, showing how reciprocal relationships can buffer health challenges. Although support did not erase symptoms, they made them less determinative for learning, thus giving better *functional* health, in line with Hubers et al.’s health definition [42].

Ahmed explained that the relationships he relied on when navigating the Norwegian society were with friends in the majority population, not fellow refugees. *‘A dead person cannot carry a dead person’* he said, explaining that peers who had fled and been traumatized like himself could not provide the support he needed.

Hassan, a single man in his late thirties, contrasted formal training with informal relationships:

*And what was important was that I did volunteer work. I worked at a second-hand store… The formal way is to go to school, the introduction program, that is, the training opportunities. And the informal way is to seek out making friends yourself*

Volunteering at a second-hand shop gave him both language practice and friendships. He described an elderly colleague as someone he could confide in and learn from, *‘To understand the society, to be active and contribute’*, he explained, highlighting reciprocity as a motivation.

Across cases, reciprocity transformed social contact into mutual exchange rather than one-sided support. In Vygotskian terms, these balanced relationships provided learning support in everyday life, often more compassionate and frequent than classroom instruction. Aligning with Arendt’s perspective [35], reciprocity also created symmetry and allowed individuals to show and be appreciated for their subjectivity.

#### 3.2.3. Trust and Companionship

Through social interactions, participants gradually developed trust and companionship that supported language learning. Lina, who wanted to move from Norway at some point, told her neighbours:

*Even if I go back to my home country someday, I will always remember you. You treated me in such a good way and took care of me, helped me when I had difficulties, not only with tasks, but with my Norwegian.*

Her gratitude illustrates how companionship created safe spaces for practice, where potential mistakes could become part of relationship-building rather than reasons for withdrawal.

Zara, struggling with severe depression, credited her learning to a teacher’s encouragement:

*She was very good and supported me all the time… When I wanted to cancel my first Norwegian exam she said, ‘No, I am absolutely positive, you have to do it. Just believe me, you will do it’.*

The teacher provided both linguistic support and emotional reassurance, ensuring challenges stayed within reach. This support prevented avoidance and generated confidence that also eased her symptoms.

Faysal, a father in his late thirties, developed close friendships through football. His teammates invited him to social events, sometimes joking about differences in faith and practice: *‘Faysal, what are you doing this weekend? Want to join us? We’re going to a nightclub, for example. Yeah, yeah, yeah, we’re just kidding…’,* knowing that he neither partied nor drank alcohol. Such humour, grounded in respect, signalled belonging. Although Faysal abstained from alcohol, his peers ensured inclusion in gatherings and respected his choices. Shared humour and accommodation reinforced companionship and motivated continued engagement, in Norwegian.

Across these cases, trust and companionship transformed relationships into supportive contexts for learning. Safe, reciprocal bonds encouraged linguistic risk-taking, perseverance, and recognition as equals, all these being conditions central to both communicative power and sustained language acquisition.

#### 3.2.4. Summary of Relational Support and Language Learning

Practical support, reciprocity, and trust fostered relationships that opened spaces for language learning and belonging. In Vygotskian terms, social interaction functioned as guided support within the Zone of Proximal Development, allowing learners to progress despite health burdens. Small successes generated confidence and further engagement, creating upward spirals of practice and belonging. From Arendt’s perspective [35], reciprocity and companionship created symmetry and belonging: in acting and speaking together as equals there was recognition and dignity, countering experiences of powerlessness. These opportunities were situational: Emerging in workplaces, volunteer arenas, and neighbourhood, where everyday activities carried meaning and shared purpose.

## 4. Discussion

This study explored how health challenges intersect SLA among Syrian refugees in Norway and how language learning can be facilitated trough relational support. Guided by reflexive thematic analysis, two main themes were generated:

(1) Learning under strain, where structural, psychosocial and embodied health burdens constrained learning, and (2) Relational Support, where practical activities, reciprocity and trust enabled participation and learning despite health challenges.

Stories of participants in this study highlight that lack of control over one’s life in the trajectory of migration were not only experienced socially but could also be embodied, in line with Sir Marmot’s viewpoints [33]. Aligning with Arendt’s perspectives [35], lack of control could be counteracted through linguistic communication and togetherness, creating symmetry and recognition. Vygotsky’s sociocultural learning theory [44] further explains the mechanism: For participants, language advanced through guided support and reciprocity within trusted interactions. Importantly, even those with health challenges were able to function and learn more in these interactions, thereby strengthening their capacity for self-management, in line with Huber et al.’s understanding of health [42]. Together, these perspectives show that learning under strain is not solely an individual cognitive challenge, but is shaped by health, power and mutuality. This resonates with classical Arab perspectives on learning. Al-Ghazali described knowledge as embodied and relational, not merely intellectual mastery, but a process that shapes both body and soul, linking learning to wellbeing [51]. Similarly, ibn Khaldun emphasized that knowledge flourishes only through social cohesion and solidarity, underscoring that education is sustained by trust and community [52,53].

This relational dimension was expressed by Ahmed, whose metaphor “A dead person cannot carry a dead person” captures the limits of support within equally traumatised refugee networks. In Vygotskian terms [44], learning depends on interaction with others who can extend one’s capacities. If all are equally constrained, such progress is difficult. For Ahmed, advances in both language and functional health relied instead on ties with majority members, who provided guidance he could not find among fellow refugees. This points to a broader pattern, where ties to the majority population create openings for language practice and societal navigation and connectedness that refugee-only networks, however supportive, are less able to provide.

The absence of social connectedness is strongly linked to a wide range of poor physical and mental health outcomes including chronic illness, depression, cognitive decline, and early mortality [54]. The link between social inclusion and learning is supported by previous research showing that SLA is enhanced in socially engaging environments [55]. Masri and Abu-Ayyash qualitatively explored SLA in 45 Syrian refugees residing in nine different countries and highlighted the importance of social interactions with native speakers [56].

Emphasizing social interaction in the context of language learning aligns with the view that language is an evolving social process, rather than a fixed system [57]. This understanding reflects how participants in this study described language learning as most meaningful in relational, trust-based settings, through volunteer work, everyday conversations, or community activities, rather than solely through formal instruction. Such social dimensions were experienced as health-supportive, promoting self-worth and belonging. However, Norwegian language policy is shaped by Einar Haugen’s ecological approach [58], which compares languages to species competing for survival [59]. According to Haugen, “language planning” plays a key role in selecting, promoting, and standardizing the dominant language across society [59]. This model reinforces the idea of language as a hegemonic force tied to nation-building, an understanding that has been critiqued for sustaining social hierarchies in which some languages, and by extension their speakers, are viewed as more ‘advanced’ than others [60]. These ideological underpinnings have concrete consequences on refugee health. Tavares and Iversen argue for the need to identify and challenge the structural discrimination embedded in second-language education [61].

In Norway, language training is not just encouraged; it is legally mandated for refugees, with non-compliance potentially leading to financial penalties [20]. Citizenship, often regarded by participants as the final marker of belonging and recognition, hinges on passing both language and civics tests [23]. Such legislation reflects an ideology that tightly ties language proficiency to national identity [62], implicitly constructing those who struggle with Norwegian as less legitimate members of society.

As Paulo Freire argued, “the whole activity of education is political in nature” [63]. Indeed, entitlement to and obligation of linguistic education for refugees following resettlement is inherently a policy matter. This becomes especially visible in the case of forced migrants, for whom language education is simultaneously a right, duty, and gateway to full societal inclusion. The current model, while promoting integration through language, risks pathologizing those who do not succeed and may obscure the structural barriers that contribute to trauma, pain, and psychological distress, which in turn complicate language acquisition.

In addition to linguistic factors, social isolation, which has detrimental effects on both physical and mental health, is the strongest predictor of heightened psychological distress [64]. In a systematic review of refugees’ psychosocial stressors post-resettlement, mental and physical health problems were found to be substantial consequences of loneliness [65]. A study of Syrian refugees in Sweden found that social strain, such as a lack of social support networks, negatively affected health-related quality of life [66]. In the context of language learning, social interactions with the majority population are particularly important. Exposure to the recipient country’s language through social interaction with the majority population was the primary driver of proficiency in a longitudinal study of seven thousand refugees in Germany [67]. It could be argued that interaction with the majority population solely through introduction programs and language training is insufficient to foster a sense of belonging, improve health, or facilitate effective language learning.

In line with Huber et al.’s conceptualization of health, improving health in this context does not necessarily imply the complete absence of symptoms or illness. Health is seen as a dynamic balance between a person’s opportunities and limitations, both of which can change over time and are influenced by social and environmental factors [42]. For instance, Ahmed’s ability to establish connections across diverse social networks supported both his language acquisition and daily functioning, despite ongoing health challenges. Thus, social interaction and support play a crucial role in fostering language development and enhancing overall health.

### 4.1. Strengths and Limitations of the Study

Despite efforts to ensure diversity within the group, the sample was specific to Syrian refugees in Norway, which may not fully capture the experiences of other refugee groups. While the main Arabic interpreter ensured effective communication, participants who conducted interviews in Norwegian may not have expressed themselves fully, particularly on complex topics such as health. The analysis was guided by theoretical frameworks, providing structure, but may also have limited alternative interpretations, despite our efforts to capture complexities beyond these frameworks. Reflexive thematic analysis is inherently subjective and influenced by the researcher’s perspective. While it provides deep engagement with the data and contributes to the depth of interpretation, the transferability of the results should be considered in light of this subjectivity. While the results are most directly applicable to similar refugee populations in Norway, they may also offer insights relevant to broader contexts. The depth of this study is a strength; however, future research with larger, more diverse samples, longitudinal data, and additional theoretical frameworks could enhance transferability.

### 4.2. Implications

Depictions of participants in this study point to the value of fostering social interactions and informal companionship with members of the majority population, where language learning occurs not in isolation but as an evolving social process. Participants who reported health challenges yet succeeded in acquiring the language often described active involvement in volunteer work or informal connections with neighbours and colleagues. Even those with limited language skills experience a sense of belonging and psychosocial support through social engagement. Therefore, promoting social inclusion and language learning in trust-based contexts should be recognized as a form of health promotion in refugee integration policies and practices. Furthermore, increased language proficiency in socially supportive settings may strengthen individuals’ health literacy, that is, their ability to access, understand, and act on health information [68].

This study adds that social inclusion, even outside paid employment, can facilitate both language acquisition and improved health. These insights suggest that creating low-threshold, trust-based arenas for interactions with the majority population is essential. Examples include joint volunteer initiatives, informal community programs, and expanded roles for civil society actors. Such approaches recognize the resourcefulness and agency of forced migrants, while also addressing the health-related consequences of isolation and marginalization.

## 5. Conclusions

This study shows how health, social relations and second language acquisition are interrelated in the everyday lives of Syrian refugees in Norway. Through a reflexive thematic analysis of participants’ accounts, language learning is understood as a socially situated process shaped by health challenges, power relations and access to meaningful interaction, rather than only by formal teaching. The results illustrate how social engagement and relational support can enable participation in society and language learning even in contexts of health-related vulnerability. With refugees’ experience at the centre, this study contributes to an empirically grounded understanding of language as a social determinant of health.

## Figures and Tables

**Table 1:** Informant demographics (N = 20).

Characteristics	Category	Number of Participants
Gender	Men	11
	Women	9
Marital status	Married	12
	Widowed/Divorced/Single	8
Migration route	Asylum seeker	12
	Quota refugee	6
	Family reunification	2
Employment/education status	Employed	5
	Homemaker	2
	Long-term sick leave	4
	Disability benefits/Retired	3
	Student	6

## Data Availability

Due to privacy and ethical restrictions, full interview transcripts are not publicly available. Anonymized excerpts supporting the findings are available from the corresponding author on reasonable request.
